# Use of student feedback to drive quality improvement (QI) in a preclinical U.S. medical school course

**DOI:** 10.1080/10872981.2019.1583968

**Published:** 2019-02-27

**Authors:** Paul S. Richman, Doreen M. Olvet, Sahar Ahmad, Latha Chandran

**Affiliations:** a Department of Medicine, Division of Pulmonary, Critical Care & Sleep Medicine, Stony Brook University School of Medicine, Stony Brook, NY, USA; b Department of Science Education, Donald and Barbara Zucker School of Medicine at Hofstra/Northwell, Hempstead, NY, USA; c Department of Pediatrics, Stony Brook University School of Medicine, Stony Brook, NY, USA

**Keywords:** Course quality improvement, horizontal integration, physiology, pathophysiology, pulmonary

## Abstract

Medical educators are continually looking for ways to enhance integrated learning and help students see how the material taught in their various courses is inter-related. . At Stony Brook School of Medicine, we embarked on a school-wide new curriculum called the Learning focused, Experiential, Adaptive, Rigorous and Novel (LEARN) curriculum and developed several integrated courses that were not based in specific departments. As part of this process, the pre-clinical (Phase-1) curriculum was shortened to 17 months to accommodate an expanded set of clinical offerings. The new structure called for teachers from different departments to lead and conduct the integrated blocks of pre-clinical courses. In this paper, we describe our discouraging experience with the first iteration of an integrated course in Cardiology, Pulmonology and Renal organ systems (CPR), and its transformation into a highly successful second iteration. This involved a systematic course quality improvement (QI) process within the context of a larger school wide curricular reform. As a result, student overall satisfaction with the course increased from 22% (28 of 127 responders) to 83% (111 of 134 responders); the mean score on a standardized NBME content exam increased by 6.7%. We report the systematic process we used to collect data from students and faculty that helped facilitate quality improvement in a key course in Phase-1 of our LEARN curriculum.

## Introduction

In recent years, a significant number of US medical schools have reduced the time devoted to foundational sciences from 24 months to 18 months or fewer [1]. In general their curricula have been designed with a view toward greater horizontal integration in organ-based courses [2], in order to deliver content in a more digestible form over a shorter timeframe [3,4].

The Stony Brook University School of Medicine (SBU SOM), moved to a 17-month pre-clinical Learning focused, Experiential, Adaptive, Rigorous and Novel (LEARN) curriculum in 2015 [5]. This involved merging the previously separate course in basic physiology with the organ systems courses, which already included pathophysiology, histology and pharmacology. However, for the combined organ-system course cardiology-pulmonary-renal (‘CPR’), this merger proved to be a disruptive change. The course received unfavorable student evaluations in the 2015 transition year. A number of students failed the course, prompting the curriculum committee to request a course quality improvement (QI) review. Here we describe the changes made to the CPR course in response to student feedback, and highlight how a robust QI process helped us enhance horizontal integration, limit excess course material and improve consistency in teaching and assessments methods.

## Methods

The 10-week CPR course is a required pre-clinical (Phase I) course at SBU SOM, directed by an overall course director, 3 clinicians who directed the individual organ segments and a basic physiologist. In both 2015 and 2016, the instructional strategies consisted of approximately 40% lectures, 20% case-based small-group seminars and 40% other learning activities including 3 objective structured clinical examination (OSCE) events, a clinical pathologic conference (CPC) exercise, team-based learning, several laboratory sessions and a few independent group-study hours. Most of the non-lecture sessions included brief graded quizzes, culminating in a customized National Board of Medical Examiners (NBME) final exam that was identical in 2015 and 2016. At the end of every course, students are required to complete a survey rating their level of agreement with 14 favorable descriptor statements about course content, teaching quality and assessment methods, using a 5-point Likert scale; for each descriptor statement, students must choose one of the following responses: Strongly Agree; Agree; Neutral; Disagree; Strongly Disagree. (Table 1). They are encouraged to provide free-text comments as well. The SOM requires every course to use this survey to conduct a QI process every 2–3 years, and present an action plan to the Curriculum Committee,

**Table 1.** Percent of students who strongly agree or agree with each of the statements below.

Student survey questions	2015 (N = 127)	2016 (N = 134)
This course had clear learning objectives	46%	92%
This course met its stated objectives	36%	90%
The teaching methods were appropriate for the stated objectives	17%	76%
Adequate time was provided to meet the learning objectives	20%	58%
The course content was relevant and of sufficient detail	38%	84%
The learning materials in this course were appropriate	25%	75%
There was good integration of basic science and clinical	51%	81%
Overall, this course was well structured	22%	69%
The evaluation methods were clear	38%	87%
The evaluation methods were applied consistently and fairly	39%	80%
I received timely feedback on my performance in this course	43%	72%
The course director was responsive to students’ concerns and needs	51%	84%
The learning environment conveyed the values of collaboration, respect and integrity	60%	87%
Overall I am very satisfied with this course	23%	83%

*P*-values were < 0.001 by the Pearson Chi Square test for all items, comparing 2015 and 2016.

After an unfavorable survey after the 2015 CPR course, the course co-directors conducted a 4-month QI review. The overall course director reviewed the Likert survey responses and tabulated the 96 free-text comments to identify any critiques that were offered by multiple students. The five course directors then met on a monthly basis to plan and implement changes to the structure of the course and the content of individual learning sessions. This resulted in plans for three overall modifications of the 2015 course:

1. Tighter faculty cooperation: the basic science and clinical faculty agreed to preview and sit in on each other’s lectures whenever possible to ensure that concepts were taught in a consistent fashion with less redundancy and overlap. As a result, the course directors decided to deliver more lectures personally and revise their slides; some guest lecturers were ‘fired’ and some extraneous material was culled from the course. Faculty preparation for small group seminars was enhanced by mandating that course directors conduct face-to-face meetings with preceptors to discuss the cases and the answers in advance. A new cardiology co-director and a new pathologist were recruited, chosen on the basis of their communication skills and responsiveness.
2. Integration of content between disciplines: For each organ system, the relevant normal physiology lectures were delivered immediately prior to the pathophysiology lectures for that topic, unlike in the previous 2015 course when the normal physiology lectures for all 3 organ-systems were delivered as a contiguous block prior to the three organ pathophysiology segments. For class sessions on topics that bridged two or more organ systems (e.g., TBLs and CPCs) two or three co-directors were asked to be present.
3. More uniform course structure and assessments: The existing small group seminars were focused more uniformly on case-based learning, with smaller numbers of students and similar types of MCQ assessment at the end of the seminar. The Renal and Pulmonary segments in 2015 had included several ‘self-study’ hours which were replaced in 2016 with structured Q&A sessions by the course directors. The total number of course hours was unchanged. A larger percent of the course grade was derived from objective MCQ questions, mostly derived from NBME sources. Subjective grading of students’ verbal responses in seminar sessions was eliminated. Course content was reviewed in detail to ensure that it corresponded well to the content of the NBME exam. Each organ system was given equal grade weight.

Aside from these modifications, no other change were made in the course’s pedagogic style or structure. After the revised 2016 course, a staff member in Office of Academic and Faculty Affairs conducted a structured interview with each of the 5 course co-directors to identify what changes had actually been implemented between 2015 and 2016. Finally, the NBME exam grades were compared between the two years.

Data were statistically evaluated using IBM SPSS Statistics (IBM Corp. Armonk, NY, USA, version 22.0). The Pearson Chi-Square test was used to compare student responses in 2015 to student responses in 2016 to the 14 Likert-scale items. Responses were categorized into 3 groups: Disagree (combining Strongly Disagree and Disagree), Neutral, and Agree (combining Strongly Agree and Agree). Data from the end-of-course evaluation survey are presented as the percent of students who Strongly Agree or Agree with each of the 14 statements (Table 1). The Student’s t-test was used to compare CPR NBME scores in 2015 to 2016. NBME final score is presented as mean ± standard deviation. *P*-values <0.05 were considered statistically significant.

## Results

Data were analyzed from 127 students in the 2015 CPR course and 134 students in the 2016 CPR course. Starting in 2015, the LEARN curriculum allotted 22% fewer instruction days to teaching the physiology and disease manifestations of the cardiology, pulmonary and renal systems (39 days vs 50 days in the past). In the 2 years prior to introducing the LEARN curriculum, on average 77% of students in the CPR course agreed with the statement, ‘Overall I am very satisfied with this course.’ Following the shortened 2015 course the percent ‘overall satisfaction’ declined to 22%.

A 4-month QI process after this unsuccessful 2015 CPR course focused on modifications in 3 broad categories, below. Interviews with the course co-directors after the 2016 course revealed that the following changes had been implemented between 2015 and 2016

### Faculty cooperation and quality

1. In 2015 only 44% of sessions were taught by the course directors. The number of ‘guest’ lecturers was reduced in 2016, such that 136 of the 171 sessions (80%) were taught by the five course co-directors. Students were encouraged to email questions to the course directors, who often shared the answers with the entire class.
2. Instructors who were perceived as being ineffective were dismissed and replaced by new instructors whose teaching materials were guided by the course co-directors.
3. Basic science and clinical instructors attended roughly 1/3 of each other’s sessions

### Integration of content between disciplines

1. In 2015, a basic scientist taught a single physiology course covering all three organ systems, followed by three separate organ-system courses that covered pathophysiology of diseases, histopathology and pharmacology. This temporal sequence was changed in 2016 such that basic physiology of each organ system was taught concurrently with instruction about disease states. This allowed material to be presented more efficiently.
2. Several learning sessions were updated to focus on the functional interaction of the three organ systems, including two OSCEs, a CPC and a team-based learning exercise
3. Roughly 10% of the 2015 course content was judged to be non-foundational and was eliminated.

### Uniform course structure and assessments

1. The style of case-based seminars was made uniform between the three organ segments
2. Graded homework assignments and quizzes were each assigned a grade weight of 1% – 2%. The quizzes were administered at the end of each seminar in an identical way throughout the course.
3. Subjectively graded assignments were eliminated. Fifty-six percent of the course grade was based on quizzes consisting of questions derived from NBME sources.

The table shows the percentage of students in each year who marked Strongly Agree or Agree with each of the 14-items of the end-of course evaluation. There was a significant difference between the 2015 and 2016 ratings for all 14 items (all *p*-values < 0.001). Over this interval, the percent of students who agreed with the statement ‘Overall I am very satisfied with this course’ increased from 22% (28 of 127 responders) to 83% (111 of 134 responders), χ^2^ = 94.7, p < 0.001. The course duration remained the same in these two years.

#### NBME scores

The final CPR NBME exam percent scores significantly improved from 77.9 (± 10.0) in 2015 to 84.4 (± 7.6) in 2016 (*t*(258) = −5.817, *p* < 0.001), results expressed as mean ± S.D.

## Discussion

In 2014–2015 the Stony Brook SOM shortened its pre-clinical curriculum to 17 months, requiring all course directors to revise their courses. The first iteration of the new CPR course did not adapt successfully to this change, leading to a perception by students that inadequate time was provided to digest the material. This led to an understandable rise in student critiques about all aspects of the course, including faculty responsiveness, unfair assessments and disorganized course format. The CPR course directors were able to successfully address these critiques the following year (2016) by revising the curricular strategy during a structured QI process, with no change in course duration. An assessment of the revised course revealed that improved student satisfaction with the 2016 CPR course was most likely due to: breaking down the previously separate courses in physiology and pathophysiology, and instead juxtaposing basic science and clinical lectures dealing with the same topic (for example, a basic scientist’s lecture on the physiology and biochemistry of pulmonary blood vessels was given immediately prior to a clinician’s lecture on pulmonary hypertension); reducing instances of redundancy and overlap in course content by encouraging course co-directors to preview and attend each other’s teaching sessions, especially those that bridged two disciplines; improving consistency of teaching by having the course directors teach nearly all sessions; culling extraneous topics from the course; ensuring a uniformly close match between the learning materials and standardized exams; eliminating subjectively graded assessments; recruiting new and enthusiastic faculty instructors who were closely supervised. Educational enhancements of this nature have been discussed and implemented at many other medical schools [6–9], though we believe our example is an instructive one.

Based on the higher satisfaction ratings, we were not surprised to find significantly higher final NBME exam scores in 2016 than 2015. Other findings were, however, unexpected. For example, in 2016 students perceived less time-pressure, even though there was no change or redistribution in the number of course hours (180 hours). This perception may have resulted from the more compact format of an integrated course and reduced extraneous content. We were also surprised to find that despite the addition of some mandatory lectures and fewer independent group-study hours, students judged teaching methods to be more appropriate in the 2016 CPR course than the 2015 course.

## Conclusions

A shortened preclinical curriculum can be a challenging disruption for medical school course directors. Despite this new curricular framework, a structured QI process can enable faculty to raise the level of student satisfaction and course grades by better integrating basic and clinical sciences, engaging course faculty in closer collaboration, and improving assessments.
